# Peripheral Blood Mononuclear Cell Expression of Cation-Chloride Cotransporter (CCC) Genes in Premenstrual Dysphoric Disorder (PMDD) across the Menstrual Cycle—A Preliminary Study

**DOI:** 10.3390/biology13060377

**Published:** 2024-05-25

**Authors:** Soojeong Cho, Fatimata Soumare, Sunni L. Mumford, Paola C. Rosas, Zarema Abrieva, John M. Davis, Ajna Hamidovic

**Affiliations:** 1 Department of Pharmacy Practice, College of Pharmacy, University of Illinois at Chicago, Chicago, IL 60612, USA; scho204@uic.edu (S.C.); fsouma2@uic.edu (F.S.); prosas@uic.edu (P.C.R.); 2 Department of Biostatistics, Epidemiology and Informatics, Perelman School of Medicine, University of Pennsylvania, Philadelphia, PA 19104, USA; sunni.mumford@pennmedicine.upenn.edu; 3 Genomics Research Core, College of Medicine, University of Illinois at Chicago, Chicago, IL 60612, USA; zarbieva@uic.edu; 4 Department of Psychiatry, College of Medicine, University of Illinois at Chicago, Chicago, IL 60612, USA; davisjm@uic.edu

**Keywords:** cation–chloride cotransporters, premenstrual dysphoric disorder, peripheral blood mononuclear cell

## Abstract

**Simple Summary:**

The efficacy and polarity of γ-aminobutyric acid-A (GABA-A) receptor-mediated transmission is modulated by four KCCs (KCC1, KCC2, KCC3, KCC4) and two NKCCs (NKCC1, NKCC2) cotransporters, which respectively extrude and accumulate intracellular chloride. Since the study of CCC gene expression in the human central nervous system (CNS) is limited, alternative peripheral models, such as peripheral blood mononuclear cells (PBMCs), which exhibit significant expression of CCCs, may be utilized to further our understanding of conditions linked to GABAergic dysfunction, such as premenstrual dysphoric disorder (PMDD). This study aimed to preliminarily investigate the cellular-level pathogenesis of PMDD by evaluating the mRNA expression of CCCs in PBMCs across the menstrual cycle.

**Abstract:**

Premenstrual Dysphoric Disorder (PMDD) is a psychiatric condition characterized by debilitating affective symptomatology in the luteal phase of the menstrual cycle. Based on the previous reports that PMDD may be related to GABAergic cellular dysfunction(s), we assessed whether cation–chloride cotransporter (CCC) gene expression across the menstrual cycle is altered in PMDD. As there are limitations in accessing the human CNS to study CCC-encoding genes, we utilized peripheral blood mononuclear cells (PBMCs) as an alternative model. We first sought to replicate previous reports characterizing CCC gene expression patterns in PBMCs of reproductive age women. We subsequently investigated potential distinct CCC mRNA expression patterns in women with PMDD. We collected blood samples across 8 menstrual cycle visits for PBMC separation/RNA extraction to study mRNA expression of four KCCs (KCC1, KCC2, KCC3, KCC4) and two NKCCs (NKCC1, NKCC2) cotransporters. We mostly replicated the earlier gene expression pattern findings, and found that the expression levels of KCC1 were significantly downregulated during the mid-follicular and periovulatory subphases of the menstrual cycle in women with PMDD. The present study shows that PBMCs is a valid model for studying GABAergic mechanisms underlying PMDD.

## 1. Introduction

Various neuropsychiatric conditions are related to the dysfunction of chloride transporters that regulate the strength of synaptic inhibition. Termed cation–chloride cotransporters (CCCs), their distinctive expression patterns play a crucial role in altering synaptic plasticity, potentially precipitating progression of disorders such as epilepsy and anxiety [1]. Thus, CCC studies may enhance our understanding of fundamental physiological functions, under both normal and pathological states [2].

CCCs control the efficacy and polarity of the γ-aminobutyric acid-A (GABA-A) receptor-mediated transmission via four K-Cl ( KCC1, KCC2, KCC3, and KCC4) and two Na-K-2Cl ( NKCC1 and NKCC2) cotransporters, which respectively extrude and accumulate intracellular chloride [3,4,5]. Investigating the expression of genes that encode CCCs in the human central nervous system (CNS) is limited due to difficulties in accessing the CNS, thereby prompting the development of alternative models, such as the peripheral blood mononuclear cells (PBMCs). Indeed, the GABAergic system in PBMCs regulates essential immune cell processes such as proliferation, and cytokine secretion [5,6]. The PBMCs appear to sufficiently express NKCC1, KCC1, KCC2, KCC3, and KCC4 cotransporters in reproductive age men and women [4], and they seem to be a sensitive model for capturing CCC gene expression changes under significant reproductive events, such as pregnancy [5]. As the study of CCC expression is still in the early stages, the present study sought to replicate the initial finding that specific CCCs are expressed in PBMCs of reproductive age women [5].

The study of CCC expression levels in PBMCs presents new opportunities for evaluating mechanisms of conditions related to GABAergic dysfunction(s), such as premenstrual dysphoric disorder (PMDD), an understudied psychiatric condition affecting between 5–8% of reproductive age women [7]. Symptom expression in PMDD is thought to be related to natural hormonal level fluctuations across the menstrual cycle, though the precise nature is not clear. Under one hypothesis, acute, but otherwise normal, hormonal shifts reflect dynamic, or cellular level, dysfunction(s) that become apparent in a form of symptom expression upon hormone and GABA-A receptor interactions that occur once some hormone concentration threshold is met [8]. As such, administration of finasteride—a 5α-reductase inhibitor—reduces the levels of the steroid hormone allopregnanolone whereby the concentration threshold of this potent neuroactive steroid hormone is not achieved, and a segment of women with PMDD experiences a relief of symptoms [9]. Allopregnanolone is normally anxiolytic, but like the other anxiolytic positive allosteric modulators of GABA-A receptors, such as barbiturates, and benzodiazepines [10], it may turn paradoxically anxiogenic in some individuals, including women with PMDD. An altered neuronal chloride homeostasis is a possible contributor to these paradoxical effects. For example, genetic mutations affecting CCC functions are associated with anxiety-like behaviors and other neurological conditions in mice as the neurotransmitter GABA turns paradoxically excitatory [11]. Furthermore, as hypothesized by Backstrom and colleagues [12], the reversal potential for chloride over the cell membrane may be shifted in women with PMDD, with elevated intracellular chloride, typically present during fetal development, producing GABA-evoked excitability in vulnerable adults.

In this study, we aimed to replicate the findings that specific CCC genes are expressed in PBMCs of reproductive age women [5]. We then preliminarily examined the mRNA expression of CCCs in PBMCs according to diagnosis (PMDD vs healthy control) across the entire menstrual cycle, focusing on the dynamic and cellular-level concept of PMDD pathogenesis (discussed above). As the GABAergic cells of the CNS are inaccessible, this proof-of-concept analysis was designed to preliminarily test whether the system is sensitive in capturing group differences. The hypothesis of this study was that unique CCC gene expression profiles will be captured in PMDD in relation to the time course of the menstrual cycle. If true, this would provide the feasibility for studying cellular-level mechanisms in PMDD using PBMCs.

## 2. Materials and Methods

### 2.1. Study Design

The design of the Premenstrual Hormonal and Affective State Evaluation (PHASE) project is described in detail in Hamidovic et al. [13]. In summary, study participants were reproductive age women who self-tested their urinary luteinizing hormone levels and kept a symptom diary [14] across two to three menstrual cycles. In the last menstrual cycle, study participants completed self-testing of their urinary luteinizing hormone using the Clearblue ovulation test and attended 8 clinic visits across the menstrual cycle according to the published protocol from the BioCycle study [15]. For example, a woman with a 28-day average from the previous two menstrual cycles was scheduled to come on days 2, 7, 12, 13, 14, 18, 22, and 27. At these visits, blood samples were collected for PBMC separation/RNA extraction (Section 2.2) of CCCs.

Clinic schedules for menstrual cycle duration are located in Table S1. These visits corresponded to (1) early follicular, (2) mid follicular, (3–5) three visits during the periovulatory subphase phase, (6) early luteal, (7) mid-luteal, and (8) late luteal subphases of the menstrual cycle.

The protocol of the project was reviewed and approved by the Institutional Review Board of The Office for the Protection of Research Subjects (OPRS) at the University of Illinois at Chicago, approval number 2018–1533. It complied with the Helsinki Declaration of 1975, as revised in 2008. Written informed consent was obtained from all study participants. PHASE is registered under NCT03862469.

### 2.2. Separation of PBMC, Isolation of Total RNA, and Real-Time Quantitative Reverse Transcription PCR

The PBMCs were collected from the human peripheral blood samples by density gradient centrifugation, following which total RNA was isolated using “Maxwell SimplyRNA Cells Kit (P/N AS1390 Promega, Madison, WI, USA)”, and analyzed with the Real-Time quantitative reverse transcription PCR as described in detail in Hamidovic et al. [16]. Reference genes importin 8 (IPO8) and TATA-binding protein (TBP) were used for normalization [5]. Normalized values, i.e., ΔCt, were computed using the geometric mean of Ct values for IPO8 and TBP as the reference assay for each sample. Real-Time PCR primer information is provided in Table S2.

### 2.3. Data Analysis

Prior to analysis, data were filtered to remove any gene that was expressed in less than 50% of the study samples. All study data were realigned according to the protocol published by Mumford et al. [15] (Table S3). Data were log-transformed as appropriate. Linear mixed effect models with random intercepts were constructed to estimate associations between gene expression levels and subphase, incorporating the repeated measures per individual. Subphases were coded as factors as following (with reference to the menstrual cycle day): early follicular (day 2), mid-follicular (day 7), periovulatory 1, 2, and 3 (days 12, 13, 14), early luteal (day 18), mid-luteal (day 22), and late luteal (day 27). Significant diagnosis by subphase interactions from linear mixed effect analyses were followed by pairwise comparisons of the estimated mean group difference by menstrual subphase.

## 3. Results

PBMCs were collected from seventeen women, of whom 4 did not display a urinary LH surge, serum LH peak, or progesterone increase on the luteal phase visits that would indicate an ovulatory cycle. We, therefore, completed the data analysis on 13 study participants—6 women with PMDD and 7 healthy women. A total of 88 samples were collected from these study participants across the menstrual cycle. On average, study participants were approximately 26 years old, with a body mass index (BMI) between normal and overweight (BMI = 25.83; Standard deviation (SD) = 3.98). These and other characteristics of study participants are summarized in Table S4.

Of the total 6 CCCs genes analyzed, KCC1, KCC2, KCC3, and KCC4 were expressed in more than 50% of samples, as indicated in Table 1. NKCC2 was expressed in only 8% of the samples, while NKCC1 was not expressed in any samples. 

The normalized average mRNA expression levels are listed in Table 2. The expression level of KCC2 was notably higher (mean value = 13.3948; SD = 1.4012) relative to KCC1, KCC3, and KCC4 genes, with mean values that ranged between −1.0667 and −0.0604.

The main effect of subphase on the expression of KCC1 was statistically significant (F_(7,56)_= 2.25; *p* ≤ 0.05) along with a significant association between subphase and KCC1 (F_(7,56)_= 3.05; *p* ≤ 0.001) (Table 3). None of the other interactions reached statistical significance. 

**Figure 1:**
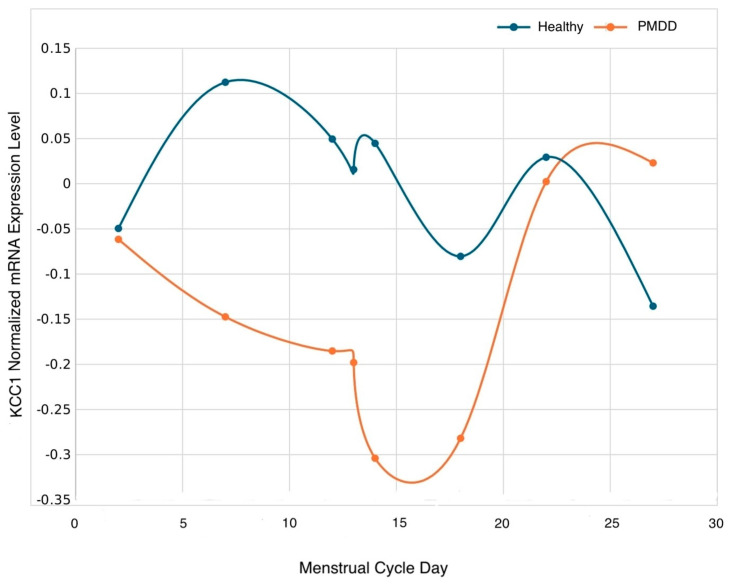
Normalized mRNA KCC1 gene trajectory according to group across the menstrual cycle on days 2 (early follicular), 7 (mid-follicular), 12 (periovulatory 1), 13 (periovulatory 2), 14 (periovulatory 3), 18 (early luteal), 22 (mid-luteal), and 27 (late luteal). Statistical differences were detected on day 7 (*p* < 0.05), and 14 (*p* < 0.05).

Pairwise comparisons of the estimated mean group difference by subphase indicated that KCC1 expression levels were higher in the healthy control relative to the PMDD group on days 7 (mid-follicular subphase) and 14 (periovulatory 3) (*p* ≤ 0.05) (Table 4 and Figure 1).

## 4. Discussion

Results of the present study indicate that KCC, but not NKCC, genes are significantly expressed in PBMCs of reproductive age women. They also preliminarily indicate that the GABAergic CCC system in PBMCs is a viable model for studying the hypothesized GABAergic cellular level dysfunction in PMDD as evidenced by a significant diagnosis by menstrual cycle subphase interaction on the mRNA expression of the KCC1 gene.

Minimal expression of NKCC1, and higher expression of KCC2 genes in PBMC consistently indicate the development of inhibitory GABAergic neurotransmission [17]. Throughout neuronal maturation, NKCC1 activity diminishes, while KCC2 activity increases in the CNS. KCC2 maintains a low intracellular chloride level, resulting in chloride influx through GABA-A receptors, which further results in hyperpolarization and neuronal inhibition. The consistent expression patterns of CCCs in both the CNS and PBMCs further support the feasibility of using PBMC as a model to investigate CCCs in neuropsychiatric conditions.

Though still in the early stages, studying the functions of CCCs in PBMCs may advance our understanding of the mechanisms underlying several neuropsychiatric disease states linked to CCCs, such as epilepsy, chronic pain, and PMDD. Indeed, CCCs are expressed in all organ systems (except for kidney specific NKCC2 and NCC), where they critically mediate neuronal and neuroendocrine signaling, as well as regulate cell volume, transepithelial ion transport, and blood pressure [17]. CCCs are expressed in PBMCs in women of reproductive age, as shown in the present study and in the earlier study by Bhandage et al. [5]. KCC1, KCC3, and KCC4 were sufficiently expressed in PBMCs of reproductive age women in both studies. However, KCC2 was undetectable, whereas NKCC1 was found to be expressed in 18 out of 19 samples from non-pregnant reproductive women in the study conducted by Bhandage et al. [5]. In the current study, we found that KCC2 was expressed in all samples, while NKCC1 was not sufficiently expressed (Table 1). These differences may be due to the test specificity (TaqMan in the present study vs SYBR green dye based testing in the Bhandage et al. study [5]). For example, for NKCC1 (SLC12A2), Taqman probe covers exon boundary 4–5, while PCR primers from Bhandage et al. [5] are within exon 4; hence, different regions were interrogated. Moreover, if traces of DNA are present, there may be additional signals, with TaqMan considered a more specific test. Regarding KCC2, given the importance of its expression on the development of neuropsychiatric disorders (reviewed in Tomita et al., 2023 [18]), the study of its expression in PBMCs may provide a novel, non-intrusive model to further the understanding of these pathological conditions.

Symptom exacerbation in women with PMDD occurs in the luteal phase, with a reliable remission in the follicular phase of the menstrual cycle. Hence, as much as it is important to study post-ovulatory triggering event(s), it is equally important to assess the milieu of the follicular phase that provides the relief of symptoms. Interestingly, expression levels of KCC1 were downregulated in the mid-follicular and periovulatory subphase of the menstrual cycle in women with PMDD relative to the healthy controls. Like KCC2, with which it shares 67% of the amino acid sequence [3], the primary role of KCC1 is lowering intracellular chloride concentration, thereby promoting the chloride influx upon the binding of GABA. Lower levels in the follicular phase would suggest higher excitation, and normalization of depressive PMDD symptomatology—a hypothesis that would need to be examined in future studies.

KCC1 is recognized as the most widely distributed isoform of KCC and is considered a fundamental membrane protein in the erythroid and lymphoid systems [19]. Considering that this study utilized PBMC as a model, it is consistent that KCC1 was prominently detected in PBMCs (i.e., lymphocytes, monocytes, and dendritic cells).

There are therapeutic agents, both investigational and FDA-approved, that target the CCCs. Bumetanide, a potent diuretic that antagonizes NKCC1, has been actively researched for the treatment of neurological and neuropsychiatric disorders such as Alzheimer’s disease, Huntington’s disease, Parkinson’s disease, Down syndrome, and epilepsy [20]. In addition, several investigative drugs, such as CLP257 and CLP290, are KCC2 stimulators under investigation for neuropathic pain [21] and refractory neonatal seizures [22]. Furthermore, the investigative drug ZT-1a indirectly influences the CCC by inhibiting SPS1-related proline/alanine-rich kinase (SPAK). Inhibition of SPAK reduces NKCC1, while stimulating KCC activity. This can potentially be applied to brain disorders associated with impaired ionic homeostasis due to ischemia [23]. Based on the preliminary finding here, the current therapeutic options (both investigational and FDA-approved) that target the CCC would be limited in treating PMDD because they mainly target NKCC1 and KCC2, not KCC1. However, the present study is a proof of concept analysis, and, as discussed in the limitation section, other positive findings may have been missed due to the issue related to power. Hence, the importance of other CCCs may be demonstrated in the future.

Provided that mRNA expression levels of KCC1 are also reduced in the relevant brain regions, such as the hippocampus where the gene is expressed [24], as they are in PBMCs, would mean that the excitatory tone is enhanced in the follicular phase in women with PMDD. Interestingly, brain GABA concentrations decrease *specifically* in the follicular phase of the menstrual cycle in women with PMDD [25], which may serve as a compensatory mechanism to maintain balance.

The findings of the present study should be assessed in light of the study limitations. This is a preliminary study, designed to permit a future structured approach to sample size determination. Although the sample size is relatively small, we controlled for a number of confounding factors, as the study participants did not take medications (including hormonal forms of birth control), have other mental illnesses, smoke, or take illicit drugs. Nonetheless, other positive findings may have been missed.

## 5. Conclusions

In conclusion, the present results extend our understanding of CCC gene expression in PBMCs of reproductive age women, and preliminarily show a significant effect of the menstrual cycle on KCC1 mRNA expression in women with PMDD. This intriguing finding provides new avenues to probe the molecular basis of PMDD.

## Figures and Tables

**Table 1:** The mRNA expression of CCCs in PBMCs.

Gene ID	Official Full Name	Number of Samples (%) with Unidentifiable Expression Levels	Removed from Analysis
SLC12A1 (NKCC2)	Solute carrier family 12 member 1	81 (92%)	Yes
SLC12A2 (NKCC1)	Solute carrier family 12 member 2	88 (100%)	Yes
SLC12A4 (KCC1)	Solute carrier family 12 member 4	0 (0%)	No
SLC12A5 (KCC2)	Solute carrier family 12 member 5	28 (32%)	No
SLC12A6 (KCC3)	Solute carrier family 12 member 6	0 (0%)	No
SLC12A7 (KCC4)	Solute carrier family 12 member 7	0 (0%)	No
IPO8	Housekeeping gene	0 (0%)	No
TBP	Housekeeping gene	0 (0%)	No

**Table 2:** Normalized average mRNA expression levels of KCCC genes.

Gene	Mean Value	SD	SE	CI
KCC1	−0.0604	0.2206	0.0235	0.0467
KCC2	13.3948	1.4012	0.1809	0.3619
KCC3	−1.6478	0.2013	0.0214	0.0426
KCC4	−1.0667	0.3439	0.0366	0.0728

**Table 3:** Predicted effects of diagnosis and subphase on mRNA gene expression.

Gene	Parameter	F value (Degrees of Freedom)	*Pr* > *F*
KCC1	Diagnosis	2.00 (1, 56)	0.1625
	Subphase	2.25 (7, 56)	0.0435 *
	Diagnosis × Subphase	3.05 (7, 56)	0.0086 **
KCC2	Diagnosis	1.23 (1, 25)	0.2770
	Subphase	0.90 (7, 25)	0.5161
	Diagnosis × Subphase	0.94 (7, 25)	0.4877
KCC3	Diagnosis	0.46 (1, 56)	0.5012
	Subphase	0.69 (7, 56)	0.6774
	Diagnosis × Subphase	1.04 (7, 56)	0.4110
KCC4	Diagnosis	0.55 (1, 56)	0.4628
	Subphase	0.85 (7, 56)	0.5541
	Diagnosis × Subphase	0.83 (7, 56)	0.5683

* *p* ≤ 0.05; ** *p* ≤ 0.001.

**Table 4:** Group (Healthy vs PMDD) contrasts according to menstrual cycle day (subphase) on KCC1 mRNA expression.

Menstrual Cycle Day (Subphase)	Estimate	Standard Error	t-Value	*p*-Value
2 (Early Follicular)	0.012	0.080	0.09	0.924
7 (Mid-Follicular)	0.259	0.127	2.05	0.045 *
12 (Periovulatory 1)	0.234	0.125	1.88	0.065
13 (Periovulatory 2)	0.213	0.138	1.55	0.127
14 (Periovulatory 3)	0.348	0.131	2.66	0.010 *
18 (Early Luteal)	0.201	0.124	1.62	0.110
22 (Mid-Luteal)	0.027	0.127	0.21	0.832
27 (Late Luteal)	−0.158	0.144	−1.10	0.276

* *p* ≤ 0.05.

## Data Availability

The data presented in this study are available on request from the corresponding author. The data are not publicly available due to preserve scientific integrity of research methodology.

## Supplementary Materials

The following supporting information can be downloaded at: https://www.mdpi.com/article/10.3390/biology13060377/s1, Table S1: Algorithm to schedule clinic visits (in days) based on average self-reported cycle length in the BioCycle Study; Table S2: RT-qPCR primers; Table S3: Algorithm for aligning the day of the luteinizing hormone (LH) surge (visit 4) on the standardized LH surge visit (O2); Table S4: Demographic characteristics of study participants. Reference [15] is cited in the Tables S1 and S3.
