# Supplementary material for: Peripheral Blood Mononuclear Cell Expression of Cation-Chloride Cotransporter (CCC) Genes in Premenstrual Dysphoric Disorder (PMDD) across the Menstrual Cycle—A Preliminary Study
Source: Biology (Basel). 2024 May 25;13(6):377. doi: 10.3390/biology13060377 (PMC11201072; doi:10.3390/biology13060377)
Supplement: Supplementary file 1 [file biology-13-00377-s001.zip › Table S1.pdf]

Table S1. Algorithm to schedule clinic visits (in days) based on average self-reported cycle length in the BioCycle Study (Mumford et al., 2011)

Average cycle length (days)	Visit 1 Menses (M)	Visit 2 Mid follicular (MF)	Visit 3 Periovulatory 1 (O1)	Visit 4 Periovulatory 2[a] (O2)	Visit 5 Periovulatory 3 (O3)	Visit 6 Early luteal (L1)	Visit 7 Mid luteal (L2)	Visit 8 Late luteal (L3)
21	2	4	5	6	7	11	15	20
22	2	4	6	7	8	12	16	21
23	2	5	7	8	9	13	17	22
24	2	5	8	9	10	14	18	23
25	2	6	9	10	11	15	19	24
26	2	6	10	11	12	16	20	25
27	2	7	11	12	13	17	21	26
28	2	7	12	13	14	18	22	27
29	2	7	13	14	15	19	23	28
30	2	8	14	15	16	20	24	29
31	2	8	15	16	17	21	25	30
32	2	9	16	17	18	22	26	31
33	2	9	17	18	19	23	27	32
34	2	10	18	19	20	24	28	33
35	2	10	19	20	21	25	29	34
