# Supplementary material for: Peripheral Blood Mononuclear Cell Expression of Cation-Chloride Cotransporter (CCC) Genes in Premenstrual Dysphoric Disorder (PMDD) across the Menstrual Cycle—A Preliminary Study
Source: Biology (Basel). 2024 May 25;13(6):377. doi: 10.3390/biology13060377 (PMC11201072; doi:10.3390/biology13060377)
Supplement: Supplementary file 1 [file biology-13-00377-s001.zip › Table S2.pdf]

Table S2. RT-qPCR primers

Gene	Assay ID	Forward Primer	Reverse Primer	Amplicon Size (bp)
SLC12A2 (NKCC1)	Hs00169032_m1	TGGGTCAAGCTGGAATAGGTC	ACCAAATTCTGGCCCTAGACTT	161
SLC12A1 (NKCC2)	Hs00165731_m1	TCAGGAGATTTGGAGGATCCC	ACCCCTAAGTAGGCAACAGTG	86
SLC12A4 (KCC1)	Hs00957122_m1	CCTCCCGTGTTTCCGGTATG	CAGGAGTCGGTCGTAAGGTTG	155
SLC12A5 (KCC2)	Hs00221168_m1	GGAAGGAAATGAGACGGTGA	TCCCACTCCTCTCCACAATC	200
SLC12A6 (KCC3)	Hs00994559_m1	GGATGTCATCGAGGACCTGAG	TCGAGCTTTCTTATGTCCGTC	82
SLC12A7 (KCC4)	Hs00986431_m1	ATCTACTTCCCTTCCGTGACC	TCTGTGCATCCTTGAGGTCC	70
TBP	Hs00427620_m1	GAGCTGTGATGTGAAGTTTCC	TCTGGGTTTGATCATTCTGTAG	117
IPO8	Hs00914057_m1	GCAAAGGAAGGGGAATTGAT	CGAAGCTCACTAGTTTTGACCC	91
