# Supplementary material for: Peripheral Blood Mononuclear Cell Expression of Cation-Chloride Cotransporter (CCC) Genes in Premenstrual Dysphoric Disorder (PMDD) across the Menstrual Cycle—A Preliminary Study
Source: Biology (Basel). 2024 May 25;13(6):377. doi: 10.3390/biology13060377 (PMC11201072; doi:10.3390/biology13060377)
Supplement: Supplementary file 1 [file biology-13-00377-s001.zip › Table S3.pdf]

Table S3. Algorithm for aligning the day of the luteinizing hormone (LH) surge (visit 4) on the standardized LH surge visit (O2) (based on the Biocycle study, Mumford et al., 2011)

Standardized cycle phase[b]									
	LH peak occurred on visit (*n*)	Menses (M)	Mid follicular (F1)	Periovulatory 1 (O1)	Periovulatory 2 (LH surge; O2)	Periovulatory 3 (O3)	Early luteal (L1)	Mid luteal (L2)	Late luteal (L3)
Early LH peak	Visit 3 (115) *Periovulatory 1*	Visit 1	Visit 2	*Missing*[a]	Visit 3	*Average* of visits 4 & 5	Visit 6	Visit 7	Visit 8
Correctly timed	Visit 4 (139) *Periovulatory 2 (LH surge)*	Visit 1	Visit 2	Visit 3	Visit 4	Visit 5	Visit 6	Visit 7	Visit 8
Late LH peak	Visit 5 (98) *Periovulatory 3*	Visit 1	Visit 2	*Average* of visits 3 & 4	Visit 5	*Missing*	Visit 6	Visit 7	Visit 8
	Visit 6 (30) *Early luteal phase*	Visit 1	*Average* of visits 3, 4 & 5	*Missing*	Visit 6	*Missing*	Visit 7	Visit 8	*Missing*
	Visit 7 (19) *Mid luteal phase*	Visit 1	*Average* of visits 3, 4 & 5	*Missing*	Visit 7	*Missing*	Visit 8	*Missing*	*Missing*
	Visit 8 (5) *Late luteal phase*	Visit 1	*Average* of visits 3, 4 & 5	*Missing*	Visit 8	*Missing*	*Missing*	*Missing*	*Missing*

[a] Missing indicates that after realignment there was no serum collection (visit) during that phase of the cycle such that the reclassified visit was set to missing.
[b] If we assume a standard 28-day cycle, the standardized cycle phases would correspond to approximately days 2, 7, 12, 13, 14, 18, 22 and 27, respectively. Alternatively, these visits could be referenced from the day of the LH surge of a 28-day cycle as: onset of menses until day -8 relative to the LH surge, day -7 to -2, day -1, day 0, day +1, day +2 to day +7, day +8 to day +11, day +12 to end of cycle.
