# Supplementary material for: Peripheral Blood Mononuclear Cell Expression of Cation-Chloride Cotransporter (CCC) Genes in Premenstrual Dysphoric Disorder (PMDD) across the Menstrual Cycle—A Preliminary Study
Source: Biology (Basel). 2024 May 25;13(6):377. doi: 10.3390/biology13060377 (PMC11201072; doi:10.3390/biology13060377)
Supplement: Supplementary file 1 [file biology-13-00377-s001.zip › Table S4.pdf]

Supplementary Table S4. Demographic characteristics of study participants		
Demographic Variable	Category	Value
Age		26.76 (4.93)
Race	White	6
	Black or African American	2
	American Indian or Alaska Native	0
	Asian	3
	Native Hawaiian or Other Pacific Islander	0
	More than one race	0
	Unknown / Do not want to specify	2
Ethnicity	Hispanic	6
	Non-Hispanic	7
	Unknown / Do not want to specify	0
Student Status	Yes	7
	No	6
Age of Menarche		11.53 (1.80)
Body Mass Index		25.83 (3.98)
Continuous variables are summarized as means (standard deviations). Data for categorical variables are presented as Ns.		
